# CRISPR-based tools for targeted transcriptional and epigenetic regulation in plants

**DOI:** 10.1371/journal.pone.0222778

**Published:** 2019-09-26

**Authors:** Joanne E. Lee, Manuela Neumann, Daniel Iglesias Duro, Markus Schmid

**Affiliations:** 1 Umeå Plant Science Centre, Department of Plant Physiology, Umeå University, Umeå, Sweden; 2 Max Planck Institute for Developmental Biology, Department of Molecular Biology, Tübingen, Germany; 3 Beijing Advanced Innovation Centre for Tree Breeding by Molecular Design, Beijing Forestry University, Beijing, People’s Republic of China; Universidad Miguel Hernández de Elche, SPAIN

## Abstract

Programmable gene regulators that can modulate the activity of selected targets *in trans* are a useful tool for probing and manipulating gene function. CRISPR technology provides a convenient method for gene targeting that can also be adapted for multiplexing and other modifications to enable strong regulation by a range of different effectors. We generated a vector toolbox for CRISPR/dCas9-based targeted gene regulation in plants, modified with the previously described MS2 system to amplify the strength of regulation, and using Golden Gate-based cloning to enable rapid vector assembly with a high degree of flexibility in the choice of promoters, effectors and targets. We tested the system using the floral regulator *FLOWERING LOCUS T* (*FT*) as a target and a range of different effector domains including the transcriptional activator VP64, the H3K27 acetyltransferase p300 and the H3K9 methyltransferase KRYPTONITE. When transformed into *Arabidopsis thaliana*, several of the constructs caused altered flowering time phenotypes that were associated with changes in *FT* expression and/or epigenetic status, thus demonstrating the effectiveness of the system. The MS2-CRISPR/dCas9 system can be used to modulate transcriptional activity and epigenetic status of specific target genes in plants, and provides a versatile tool that can easily be used with different targets and types of regulation for a range of applications.

## Introduction

One of the most common approaches for investigating gene function is testing what happens when the gene of interest is misexpressed, usually through transgenic overexpression or through gene knockout or knockdown. While these techniques are often very informative, they do have some limitations; for example, they cannot easily be used to study the impact of epigenetic marks such as DNA methylation or histone modification on individual genes, and it can be difficult to misexpress multiple genes simultaneously, which may be desirable when studying a gene family or a series of steps in a biosynthetic pathway.

An alternative method for modifying gene expression is to use programmable synthetic regulators that act *in trans* to modulate transcriptional activity or epigenetic status at a chosen target site. This technique was initially developed using Zn-finger and transcription activator-like effector (TALE) proteins, which interact with DNA in a sequence-specific manner and can be engineered to bind specific sites of interest in the genome. When fused to an appropriate effector domain, the proteins can alter the activity of the target gene, either through direct interaction with transcriptional machinery or by modifying the epigenetic landscape in that region. Such constructs have been successfully used to activate and repress transcription and to modify epigenetic marks in a variety of organisms including *Arabidopsis thaliana* (Arabidopsis), tobacco and mammalian cells (reviewed in [1] and [2]). However, the fact that the DNA-interacting regions of Zn-finger and TALE proteins must be individually engineered for each target sequence tends to make them laborious and difficult to generate, and impractical for targeting multiple genes simultaneously.

**Fig 1:**
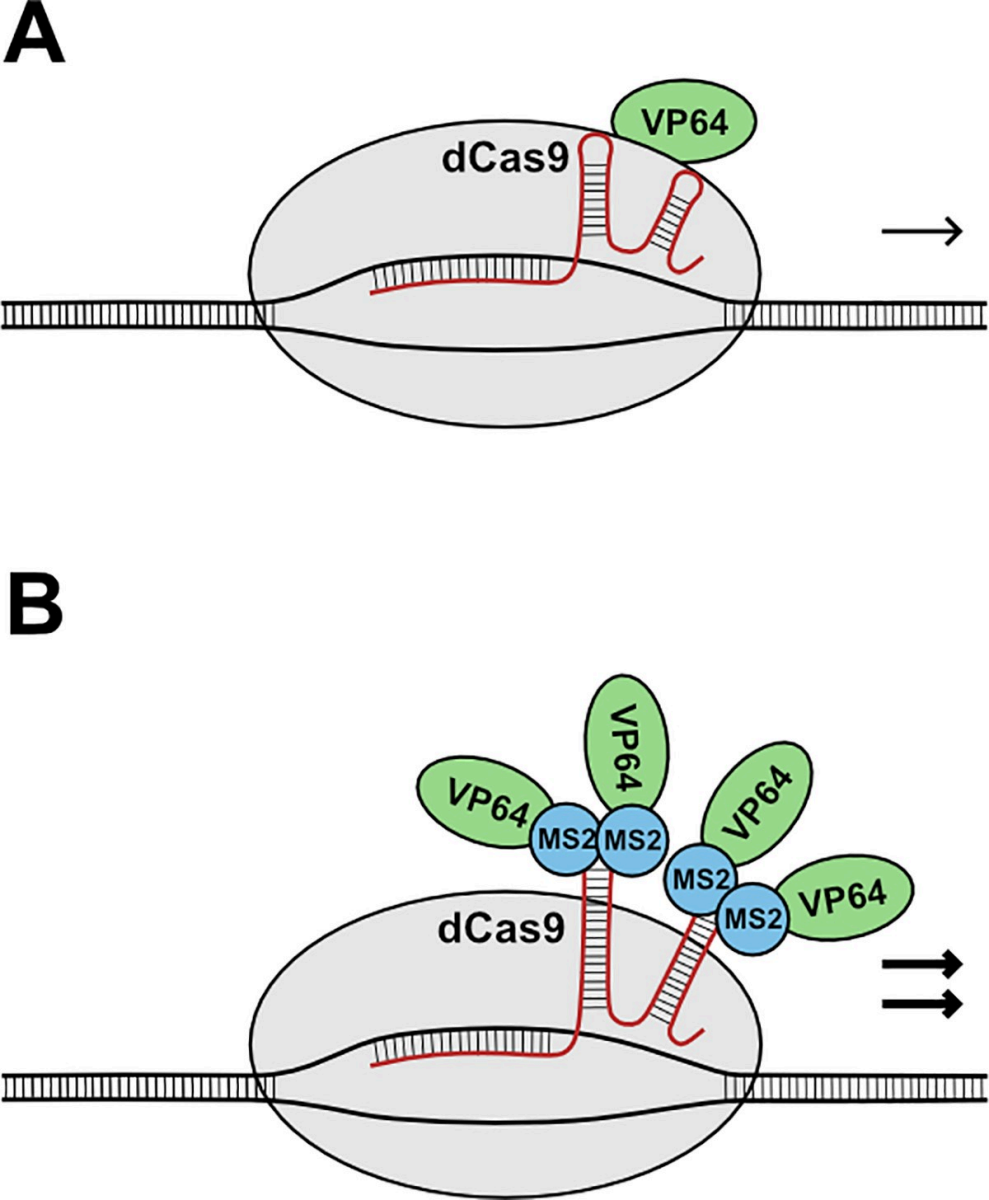
Schematic of CRISPR/dCas9-mediated targeted gene regulation. (**A**) The dCas9 protein is directed to specific sites in the genome as determined by complementary base-pairing between DNA and the sgRNA. Effector domains such as the transcriptional activator VP64 can be fused to the dCas9 protein to modulate the activity of neighbouring genes. (**B**) In the MS2 system, effector domains are recruited to dCas9 in the form of MS2-effector fusions, which bind as dimers to RNA hairpin aptamers engineered into the sgRNA. This has been shown to produce a stronger level of modulation compared to direct fusion [4, 5].

A simpler and potentially much easier approach is offered by the clustered regularly interspaced palindromic repeats (CRISPR)/Cas9 system, in which the Cas9 nuclease complex is directed to specific sites in the genome as determined by complementary base-pairing between the DNA and a short single guide RNA (sgRNA). A nuclease-dead variant of the Cas9 protein (dCas9) linked to an effector domain can be used as a synthetic regulator in similar fashion to Zn-finger and TALE proteins (Fig 1A), with the added benefits that sgRNAs are much simpler to design and synthesise than DNA-interacting protein domains, and multiple sgRNAs targeting different DNA sequences can easily be expressed from a single construct to regulate several different genes at once (multiplexing). The utility of CRISPR/dCas9 for this purpose has been widely recognised, and dCas9-based regulators of various types have been employed in numerous experimental systems (reviewed in [1] and [3]).

One limitation of CRISPR-based systems for targeted gene regulation is that direct fusions of effector domains to dCas9 often produce only a low level of modulation, possibly due to steric hindrance, as dCas9 is a large protein of approximately 160 kDa. Various modifications have therefore been developed to increase the efficiency of regulation, such as translational fusion between oligomerized *Xanthomonas* TALEs and VP128 [6] or the MS2 system of Konermann *et al*. [4], in which the effector domain is fused not to dCas9 but to the bacteriophage coat protein MS2. Dimerised MS2-effector fusion proteins recognise and bind an RNA hairpin aptamer that can be engineered into the sgRNA at two different locations, so that effectors are recruited to dCas9 via protein-RNA interaction rather than by direct fusion (Fig 1B). This strategy is designed to enable the recruitment of multiple copies of an effector domain to a single dCas9 protein, and may also improve the positioning of the effector domains in relation to dCas9. The MS2 system consistently amplified the level of VP64-mediated gene activation in mammalian cells compared to direct fusion [4], and was also recently shown to be effective for boosting transcriptional activation in plants [5].

Here, we describe the development and validation of an MS2-CRISPR/dCas9 system for targeted regulation of transcriptional activity and epigenetic status in plants. Vectors were constructed using the GreenGate cloning system [7], which is based on the Golden Gate method [8] and facilitates rapid cloning of different combinations of promoters, effector domains and sgRNAs, and proof-of-concept experiments were carried out using the flowering time gene *FLOWERING LOCUS T* (*FT*) as a target. A range of effector domains were successfully used to modulate *FT* transcriptional activity and epigenetic status, as assessed by changes in flowering time, *FT* transcript levels and histone modification levels, thus demonstrating the feasibility of our approach. This system can easily be applied to other gene targets and types of regulation, and should be of particular use for modulating the activity of multiple genes simultaneously.

## Results

### Construction of the MS2-CRISPR/dCas9 vector toolbox

**Fig 2:**
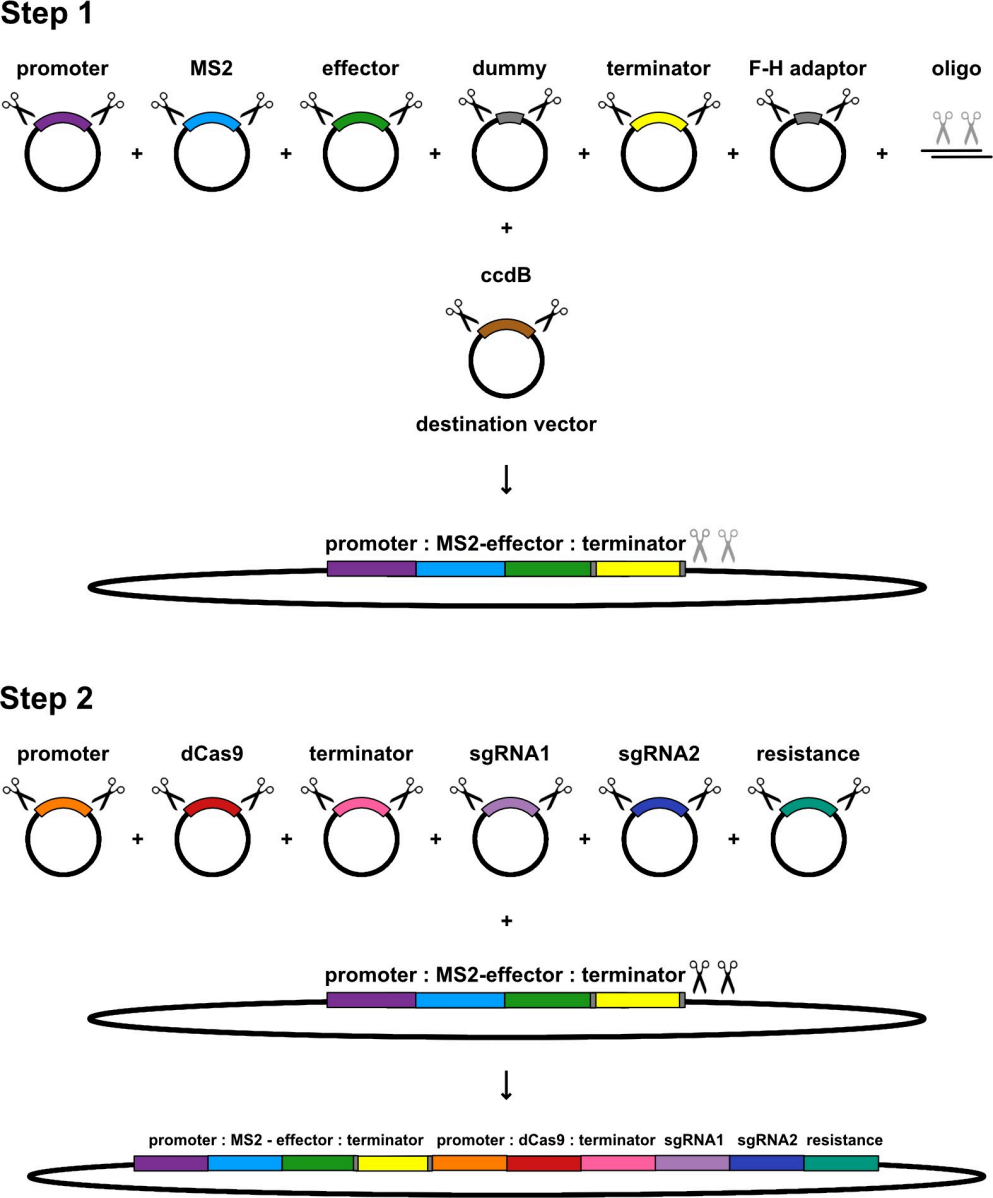
Assembly of MS2-CRISPR/dCas9 expression constructs. All GreenGate entry vectors contain an insert module flanked by *Bsa*I restriction sites (black scissors), which can be cloned into a binary expression vector in a specific order based on the overhangs generated from *Bsa*I digestion. In the first cloning step, modules containing a promoter, *MS2* and effector coding sequences, and terminator are inserted into a destination vector in place of a counter-selectable *ccdB* gene, along with an oligonucleotide duplex that contains two internal *Bsa*I recognition sites (grey scissors). These sites are initially protected from digestion by cytosine methylation, which is lost following transformation and replication in bacteria. The unmethylated construct is then used as the destination vector in a second cloning step, in which a promoter, *dCas9* coding sequence, terminator, sgRNAs and plant resistance cassette are inserted downstream of the *MS2-effector* cassette to create the final expression construct. Entry vectors containing short ‘dummy’ or adaptor sequences are used in positions where no specific module is desired, to provide the correct overhangs for plasmid assembly. This procedure can be modified to include additional modules by re-using the methylated oligonucleotide duplex to carry out further rounds of cloning.

We generated all of our MS2-CRISPR/dCas9 expression constructs using the Golden Gate-based GreenGate cloning system [7], which allows rapid assembly of plant binary expression constructs from a library of modular, pre-cloned entry vectors. Individual modules consisting of promoters, coding sequences, etc. are initially cloned into a set of entry vectors that generate different overhangs when digested with the type IIS restriction enzyme *Bsa*I. These modules can be inserted into a binary expression vector in a specific order based on the complementarity between overhang sequences, in a single cloning reaction consisting of repeated cycles of digestion and ligation. The system is fast, easy-to-use, and highly flexible, as it allows different promoters, effector domains and sgRNAs to be combined as desired. We followed a two-step cloning procedure, in which first an expression cassette encoding the MS2-effector fusion is inserted into a binary vector, followed in the second step by the *dCas9* cassette, sgRNAs and plant resistance cassette (Fig 2). The procedure can be reduced to one step, if one of our intermediate vectors containing an already-assembled *MS2-effector* cassette is used, or extended with additional steps to include extra modules such as more sgRNAs or additional *MS2-effector* cassettes. All of the entry and intermediate vectors generated for this study were deposited with Addgene for the use of the community (S1 Table).

We initially tested the MS2-CRISPR/dCas9 system in Arabidopsis using the strong transcriptional activator VP64 as the effector domain fused to a nuclear-targeted MS2, and the floral regulatory gene *FT* as the sgRNA target. *FT* is normally expressed in phloem companion cells in response to long photoperiods, and is a key determinant of flowering time in long day (LD) conditions [9]. Any change in *FT* expression should therefore cause a corresponding shift in flowering time, thus providing a simple phenotypic read-out for the effectiveness of our synthetic regulators.

We generated four expression constructs in which the *MS2-VP64* and *dCas9* coding sequences were placed under the control of different combinations of the ubiquitously active cauliflower mosaic virus (CaMV) *35S* promoter and the phloem-specific *SUC2* promoter [10] (*35S*:*MS2-VP64*/*35S*:*dCas9*, *35S*:*MS2-VP64*/*pSUC2*:*dCas9*, *pSUC2*:*MS2-VP64*/*35S*:*dCas9* and *pSUC2*:*MS2-VP64*/*pSUC2*:*dCas9*; Table 1). In addition to the *MS2-VP64* and *dCas9* expression cassettes, each of the constructs also contained two sgRNAs, both modified with MS2-binding aptamers and targeted against neighbouring sites in the *FT* proximal promoter (sgRNA-FT-A and sgRNA-FT-B), and a Basta resistance cassette for selection in plants.

**Table 1:** MS2-CRISPR/dCas9 expression constructs used in this study.

Plasmid	Description	Effector domain	Effector function
pJL005	*35S*:*MS2-GFP*:*tRBCS* *35S*:*dCas9*:*tRBCS* *sgRNA-FT-A* *sgRNA-FT-B* *35S*:*BastaR*:*t35S*	GFP	Fluorescent marker (negative control)
pMH211	*35S*:*MS2-VP64*:*tRBCS* *35S*:*dCas9*:*tRBCS* *sgRNA-FT-A* *sgRNA-FT-B* *pMAS*:*BastaR*:*tMAS*	VP64	Transcriptional activator
pMH212	*35S*:*MS2-VP64*:*tRBCS* *pSUC2*:*dCas9*:*tRBCS* *sgRNA-FT-A* *sgRNA-FT-B* *pMAS*:*BastaR*:*tMAS*		
pMH213	*pSUC2*:*MS2-VP64*:*tRBCS* *35S*:*dCas9*:*tRBCS* *sgRNA-FT-A* *sgRNA-FT-B* *pMAS*:*BastaR*:*tMAS*		
pMH214	*pSUC2*:*MS2-VP64*:*tRBCS* *pSUC2*:*dCas9*:*tRBCS* *sgRNA-FT-A* *sgRNA-FT-B* *pMAS*:*BastaR*:*tMAS*		
pJL011	*35S*:*MS2-SRDX*:*tRBCS* *35S*:*dCas9*:*tRBCS* *sgRNA-FT-A* *sgRNA-FT-B* *pMAS*:*BastaR*:*tMAS*	SRDX	Transcriptional repressor
pJL044	*35S*:*MS2-p300*:*tRBCS* *35S*:*dCas9*:*tRBCS* *sgRNA-FT-A* *sgRNA-FT-B* *pMAS*:*BastaR*:*tMAS*	*Homo sapiens* p300 core domain	H3K27 acetyltransferase
pJL049	*35S*:*MS2-G9a*:*tRBCS* *35S*:*dCas9*:*tRBCS* *sgRNA-FT-A* *sgRNA-FT-B* *pMAS*:*BastaR*:*tMAS*	*Homo sapiens* G9a SET domain	H3K9 methyltransferase
pJL050	*35S*:*MS2-KYP*:*tRBCS* *35S*:*dCas9*:*tRBCS* *sgRNA-FT-A* *sgRNA-FT-B* *pMAS*:*BastaR*:*tMAS*	Arabidopsis KYP SET domain	H3K9 methyltransferase

All coding sequences originating from non-plant organisms were codon-optimised for Arabidopsis. *tRBCS* = terminator of Rubisco gene (from pea); *BastaR* = Basta resistance gene; *t35S* = terminator of CaMV *35S*; *pMAS* = promoter of mannopine synthase; *tMAS* = terminator of mannopine synthase.

To check whether the physical presence of the dCas9 complex has any effect on gene expression in the absence of a transcriptional effector, we also cloned a negative control construct using the transcriptionally inert green fluorescent protein (GFP) in place of VP64, with *35S* promoters driving expression of both *MS2-GFP* and *dCas9*, and the same *FT*-targeting sgRNAs as above.

### Targeted upregulation of FT by MS2-VP64

**Fig 3:**
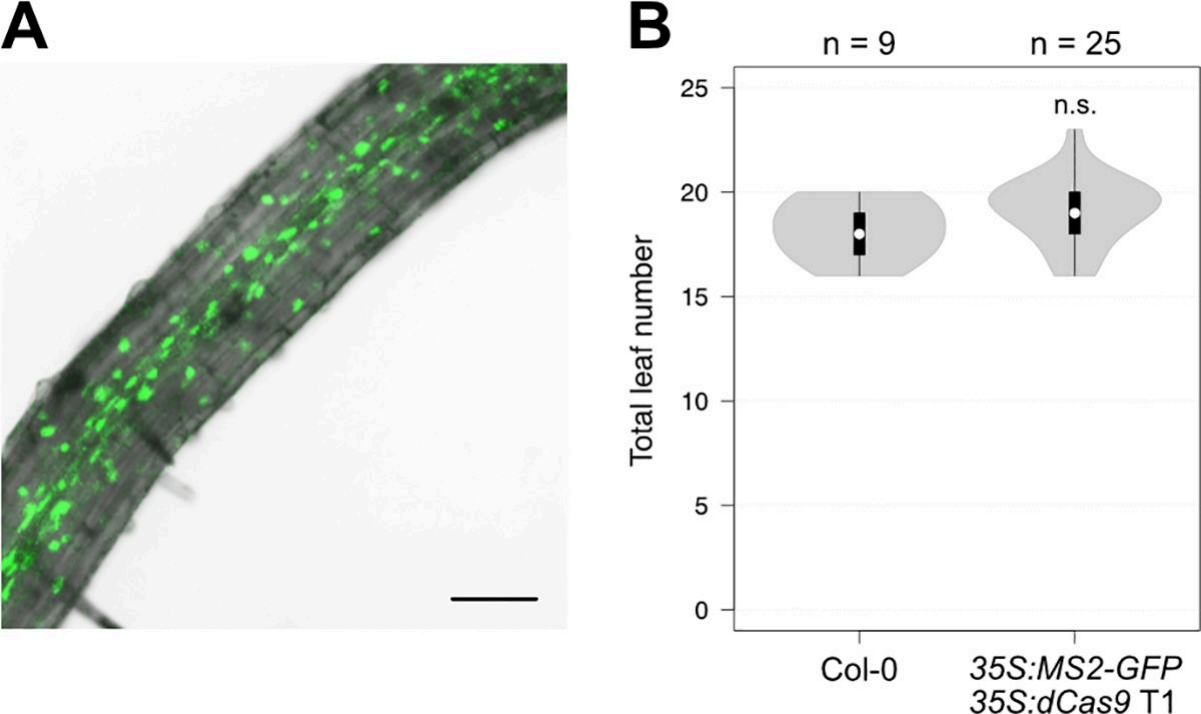
The dCas9 complex does not affect flowering time when associated with MS2-GFP. (**A**) MS2-GFP fluorescence was detected in the nuclei of root cells in homozygous *35S*:*MS2-GFP* T3 plants. Image shows overlay of GFP fluorescence on bright field. Bar = 100 μm. (**B**) Flowering time of wild type Col-0 and *35S*:*MS2-GFP* T1 plants grown in LDs. Violin plots represent density estimates of data, with white circles showing the median, black bars showing the interquartile range, and whiskers extending to data points up to 1.5 times the interquartile range from the first and third quartiles. Numbers of plants are shown above the chart, and significant difference from Col-0, calculated using Student’s t-test, is indicated above the violin plot. n.s. = not significant.

The *MS2-GFP* and *MS2-VP64* constructs were transformed into wild type Arabidopsis, accession Col-0, and flowering time was measured in LD-grown transgenic plants. The *MS2-GFP* negative control plants displayed nuclear fluorescence, confirming that the fusion protein was correctly expressed and localised, but the T1 population did not show any change in flowering time compared to wild type (Fig 3), indicating that the dCas9 complex does not itself affect transcriptional activity at the *FT* promoter.

**Fig 4:**
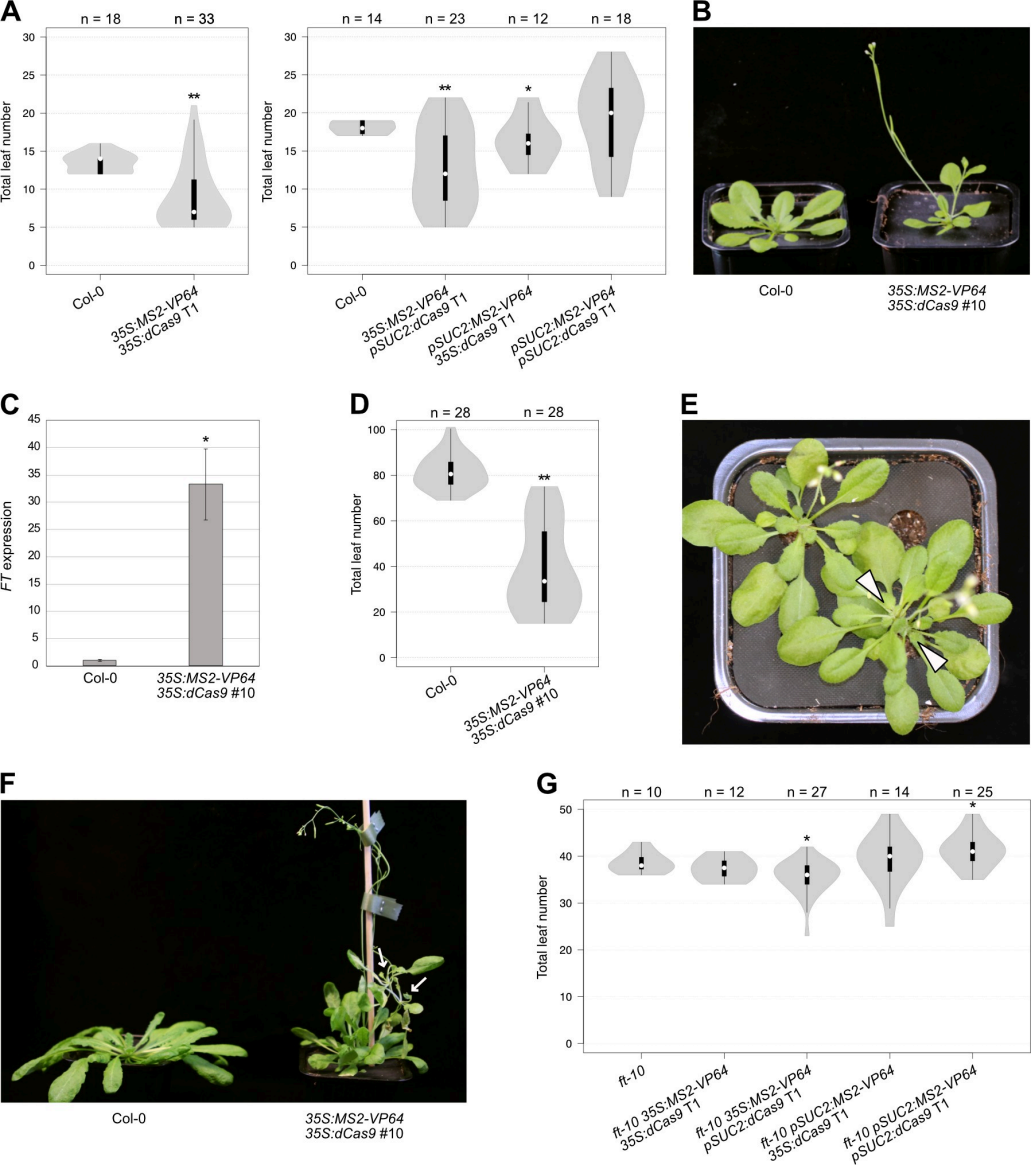
Targeted regulation of *FT* by MS2-VP64/dCas9 leads to increased *FT* expression and early flowering. (**A**) Flowering time of wild type Col-0 and *MS2-VP64/dCas9* T1 plants grown in LDs. (**B**) Phenotypes of 4-week-old Col-0 and homozygous *35S*:*MS2-VP64*/*35S*:*dCas9* T3 plants grown in LDs. (**C**) Expression of *FT* in 6-day-old Col-0 and homozygous *35S*:*MS2-VP64*/*35S*:*dCas9* T3 plants grown in LDs. Whole seedlings were harvested at Zeitgeber time (ZT) 15, and transcript levels were measured by qPCR, normalised against *TUBULIN2* (*TUB2*) and shown relative to Col-0. Values are the mean of three biological replicates ± standard error. Significant difference from Col-0 was calculated using Student’s t-test. * = P < 0.05. (**D**) Flowering time of Col-0 and homozygous *35S*:*MS2-VP64*/*35S*:*dCas9* T3 plants grown in SDs. (**E, F**) Phenotypes of (**E**) 7- and (**F**) 8-week-old homozygous *35S*:*MS2-VP64*/*35S*:*dCas9* T3 plants grown in SDs. Many of the SD-grown transgenic plants developed two rosette-producing meristems at ground level, (white arrowheads in **E**), while some individuals formed aerial rosettes on the bolting inflorescence stem (white arrows in **F**). Single cauline leaves were never observed on plants of either phenotype. (**G**) Flowering time of untransformed *ft-10* mutants and *ft-10 MS2-VP64/dCas9* T1 plants grown in LDs. Violin plots in (**A**), (**D**) and (**G**) represent density estimates of data, with white circles showing the median, black bars showing the interquartile range, and whiskers extending to data points up to 1.5 times the interquartile range from the first and third quartiles. Numbers of plants are shown above the charts, and significant differences from controls, calculated using Student’s t-test, are indicated above the violin plots. ** = P < 0.001, * = P < 0.05.

Successful upregulation of *FT* by MS2-VP64 was expected to cause an early flowering phenotype, and this was indeed the case for three of the four *MS2-VP64* constructs (Fig 4A). The strongest effects were observed in *35S*:*MS2-VP64*/*35S*:*dCas9* plants, with 24 out of 33 T1 individuals (73%) flowering earlier than any of the wild type plants, including several that produced only five leaves before flowering. The *35S*:*MS2-VP64*/*pSUC2*:*dCas9* and *pSUC2*:*MS2-VP64*/*35S*:*dCas9* T1 populations showed more moderate shifts in flowering time but still contained a majority of individuals (65% and 58%, respectively) that flowered earlier than the wild type controls, while no significant change was detected for plants carrying the *pSUC2*:*MS2-VP64*/*pSUC2*:*dCas9* construct. These results suggest that MS2-VP64 is effective at activating premature *FT* expression in a large proportion of transformed plants, provided that the strong *35S* promoter is used for at least one of the expression cassettes. Strongly early flowering individuals were all morphologically similar, regardless of which specific construct they carried, with reduced stature and leaf size compared to wild type (Fig 4B), consistent with the phenotype that has been reported for *35S*:*FT* plants [11].

We confirmed that the *35S*:*MS2-VP64*/*35S*:*dCas9* early flowering phenotype was maintained in the T2 generation in three independent lines (S1 File), and selected one of these lines to continue for further characterisation. Quantitative PCR (qPCR) analysis was performed in young T3 plants to measure *FT* expression, and, consistent with the flowering time results, we found that *FT* was strongly and significantly upregulated in transgenic seedlings compared to wild type controls (Fig 4C). The early flowering phenotype was retained when T3 plants were grown in short day (SD) conditions (Fig 4D), as expected for plants overexpressing *FT*, and SD-grown *35S*:*MS2-VP64*/*35S*:*dCas9* plants also displayed double rosette and aerial rosette phenotypes that were never observed when the plants were grown in LDs (Fig 4E and 4F). The same phenotypes were observed in SD-grown *35S*:*MS2-VP64*/*pSUC2*:*dCas9* plants, but not *pSUC2*:*MS2-VP64*/*35S*:*dCas9* or *pSUC2*:*MS2-VP64*/*pSUC2*:*dCas9* plants.

To check that the early flowering phenotype of the *MS2-VP64* plants was specifically caused by changes in *FT* regulation and was not due to off-target effects or insertional artifacts, the four constructs were also transformed into an *ft-10* mutant background. In the absence of functional FT, most of the transgenic plants flowered at approximately the same time as untransformed *ft-10* controls (Fig 4G). The *ft-10 35S*:*MS2-VP64*/*pSUC2*:*dCas9* T1 plants flowered slightly earlier than *ft-10* mutants on average, and the *ft-10 pSUC2*:*MS2-VP64/pSUC2*:*dCas9* plants slightly later, but overall these plants still closely resembled untransformed *ft-10* mutants in their flowering time. These results demonstrate that the *MS2-VP64* constructs trigger early flowering specifically through upregulation of the intended target gene *FT*, and that the MS2-CRISPR/dCas9 system is an effective method for targeted modulation of gene activity in plants.

### Modulation of FT with different MS2-effector fusions

Following the successful use of *MS2-VP64* constructs to upregulate *FT* expression, we generated a series of additional MS2-effector fusions to test whether the system is similarly effective for other types of gene regulation. The effectors that we chose were the SRDX transcriptional repressor motif [12]; the catalytic core domain of *Homo sapiens* p300, an H3K27 histone acetyltransferase that is associated with gene activation [13]; the catalytic Su(var)3-9, Enhancer-of-zeste and Trithorax (SET) domain of *Homo sapiens* G9a, an H3K9 histone methyltransferase associated with gene silencing [14]; and the SET domain of KRYPTONITE (KYP), an H3K9 methyltransferase from Arabidopsis [15] (Table 1). The three histone modifiers have all previously been used for CRISPR- or TALE-guided histone modification in animal systems and are thus known to retain functionality when fused to other proteins [16–19]. For these constructs, we again used *FT* as the regulatory target, and *35S* was used as the promoter for both *MS2-effector* and *dCas9* expression cassettes.

**Fig 5:**
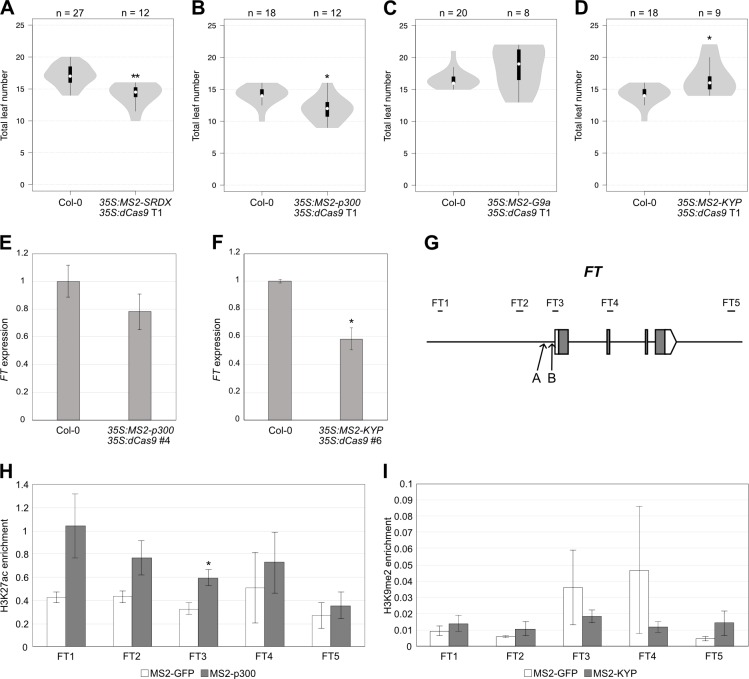
Different MS2-effector fusions regulate *FT* with varying levels of effectiveness. (**A-D**) Flowering time of wild type Col-0 and (**A**) *MS2-SRDX*, (**B**) *MS2-p300*, (**C**) *MS2-G9a* and (**D**) *MS2-KYP* T1 plants grown in LDs. Violin plots represent density estimates of data, with white circles showing the median, black bars showing the interquartile range, and whiskers extending to data points up to 1.5 times the interquartile range from the first and third quartiles. Numbers of plants are shown above the charts, and significant differences from Col-0, calculated using Student’s t-test, are indicated above the violin plots. ** = P < 0.001, * = P < 0.05. (**E, F**) Expression of *FT* in (**E**) 8-day-old Col-0 and homozygous *MS2-p300* T3 plants, and (**F**) 12-day-old Col-0 and homozygous *MS2-KYP* T3 plants grown in LDs. Whole seedlings were harvested at ZT 15, and transcript levels were measured by qPCR, normalised against *TUB2* and shown relative to Col-0. Values are the mean of three biological replicates ± standard error. Significant differences from Col-0 were calculated using Student’s t-test. * = P < 0.05. (**G**) Schematic of the *FT* locus, showing the positions of the sgRNA target sites, A and B, and the regions that were amplified to measure histone mark enrichment. Grey boxes represent the *FT* coding sequence and white boxes represent untranslated regions. (**H, I**) Enrichment of (**H**) H3K27ac and (**I**) H3K9me2 in homozygous *MS2-p300* and *MS2-KYP* T3 plants, respectively. ChIP experiments were carried out using chromatin from homozygous 15-day-old seedlings grown in LDs. Enrichment of histone marks was measured by qPCR, and data were normalised against either the highly transcribed *RBCS1A* gene (**H**) or the Cinful-like retrotransposon T5L23.29 (**I**). Values are the mean of three biological replicates ± standard error. Significant differences from *MS2-GFP* were calculated using Student’s t-test. * = P < 0.05.

We transformed the constructs into plants and analysed the flowering time of the transgenic populations, and observed varying levels of effectiveness for the different constructs. MS2- SRDX was expected to cause late flowering through repression of *FT* expression, but this was not observed in either the T1 or T2 generations (Fig 5A, S1 File). H3K27 acetylation by MS2-p300 was expected to cause early flowering, and this was indeed observed in the T1 population, although the effect was weak and not statistically significant in the T2 lines that we examined (Fig 5B, S1 File). The two H3K9 methyltransferase constructs were expected to cause late flowering phenotypes; while both the *MS2-G9a* and *MS2-KYP* T1 populations flowered later than wild type plants on average (Fig 5C and 5D), the difference was only statistically significant for *MS2-KYP*. As G9a and KYP share the same enzymatic function and the *MS2-KYP* construct appeared to be slightly more effective, we proceeded with *MS2-KYP* only, and confirmed that the late flowering phenotype was maintained in the T2 generation (S1 File).

Both the *MS2-p300* and *MS2-KYP* histone-modifying constructs were effective at altering flowering time, so we carried out further molecular characterisation of T3 lines to verify their effects on *FT*. We did not detect any increase in *FT* expression in *MS2-p300* plants compared to wild type (Fig 5E), but the *MS2-KYP* plants showed a statistically significant decrease, consistent with their late-flowering phenotype (Fig 5F). We also performed chromatin immunoprecipitation (ChIP) to measure the levels of histone acetylation and methylation around the target site. Regions in the *FT* promoter were enriched in H3K27 acetylation (H3K27ac) by approximately 2-fold in *MS2-p300* plants compared to *MS2-GFP* controls (Fig 5H), showing that targeted modification of histone marks was successful in this line even though the effects on *FT* expression and flowering time were minor. *MS2-KYP* plants, on the other hand, did not show any significant enrichment in H3K9 dimethylation (H3K9me2) at the *FT* locus (Fig 5I). Nonetheless, these combined results show that the MS2-CRISPR/dCas9 system can be successfully used with a range of different effectors to regulate both transcriptional and epigenetic status at a specific locus of interest.

## Discussion

### Effectiveness of the MS2-CRISPR/dCas9 system for different types of gene regulation

We developed a modified version of the CRISPR/dCas9 system to use for targeted gene regulation in plants, and validated our system in Arabidopsis using the flowering time regulator *FT* as a target. MS2-mediated recruitment of the strong transcriptional activator VP64 to the *FT* promoter resulted in strong upregulation of *FT* and a corresponding acceleration of flowering (Fig 4), demonstrating the basic effectiveness of the system. We observed the strongest effects when the highly and ubiquitously active *35S* promoter was used to drive expression of both *MS2-VP64* and *dCas9*, even though *pSUC2* has previously been shown to trigger early flowering with similar or higher efficiency than *35S* when driving *FT* expression directly [20]. This suggests that the MS2-CRISPR/dCas9 system may require higher levels of expression compared to traditional transgenic misexpression constructs, presumably because the MS2-VP64 fusion protein must overcome repression by native regulatory factors that are also present at the *FT* promoter. However, the *35S*:*MS2-VP64/pSUC2*:*dCas9* plants were also strongly early flowering, more so than the *pSUC2*:*MS2-VP64/35S*:*dCas9* plants, suggesting that high level expression is more important for the *MS2-effector* cassette than for the *dCas9* cassette. Efficient tissue-specific or conditional regulation of a target gene may therefore be possible using only a weak promoter for *dCas9*, as long as *35S* or a similarly strong promoter is used for the *MS2-effector* expression cassette.

In addition to the expected phenotypes of early flowering and reduced stature, we also observed double rosette and aerial rosette phenotypes when *35S*:*MS2-VP64/35S*:*dCas9* plants were grown in SD conditions (Fig 4E and 4F). Similar phenotypes have previously been reported for several other genotypes with altered flowering time regulation [21–25], but not for *35S*:*FT* plants, suggesting that the SD phenotypes arise from interplay between MS2-VP64 and native regulators at the *FT* promoter, as opposed to *FT* overexpression *per se*. As the activity of native regulators varies across cell types and developmental stages, this may produce more complex patterns of expression than are found in traditional transgenic overexpressors, thus giving rise to novel phenotypes.

Repression of *FT* by MS2-SRDX was surprisingly less effective than MS2-VP64-mediated upregulation (Fig 5A), even though the SRDX motif is known to have strong repressor activity and is regularly used in chimeric repressors [12, 26–30]. We speculate that this is due to strong activation of *FT* by the photoperiod pathway counteracting any repressive effects of the SRDX domain, and that a late flowering phenotype might be observed if the plants were grown under different conditions such as cooler ambient temperature, which is known to lower *FT* expression [31] and is sometimes used to reveal or enhance weak flowering time phenotypes [28, 32]. It is also possible that stronger repression might be observed using a different effector such as 3 x SRDX, which has previously been used to achieve targeted repression in a CRISPR-guided system [30], or different target genes that are less tightly regulated than *FT*. In general, we expect that the efficiency of MS2-CRISPR/dCas9-mediated regulation will vary widely depending on the specific gene target(s) and interplay between the chosen effector and the native regulatory environment.

The histone-modifying *MS2-p300* and *MS2-KYP* constructs had detectable but mild effects on *FT* regulation and flowering time (Fig 5). This may be because chromatin regulation generally relies on the concerted action of multiple factors, and this is particularly the case for *FT*, as a key floral regulator that integrates signals from multiple pathways and is known to be epigenetically regulated by a variety of histone-modifying enzymes with which the MS2 constructs must compete [33]. Concurrent use of sequentially-acting histone modifiers may therefore be required to overcome the effects of native epigenetic regulators and achieve strong regulation of target genes. This could potentially be accomplished using our system, by extending the GreenGate cloning procedure to include additional *MS2-effector* cassettes with different histone modifying domains in the same binary expression vector. Nonetheless, the modest changes in flowering time and observed increase in H3K27 acetylation at the *FT* promoter in *MS2-p300* plants show that targeted histone modification can be effective using the MS2-CRISPR/dCas9 system, even with only one type of histone modifier. Other groups have recently demonstrated Zn-finger- and CRISPR-based tools for targeted DNA methylation and demethylation in plants [34, 35], and targeted histone modifiers like the ones described here will make another useful addition to the suite of tools available for investigating and manipulating epigenetic regulation in plants.

### Potential applications of the MS2-CRISPR/dCas9 system

The MS2-CRISPR/dCas9 system can potentially provide a more nuanced view of gene function than the traditional methods of misexpression and gene knockout, while also offering possibilities for investigating the mechanisms of gene regulation and for studying groups of genes at the same time. While a variety of other CRISPR-based systems for plant gene modulation have previously been described [5, 30, 35–38], these generally rely on direct dCas9-effector fusions and/or provide limited options in the selection of promoters and effector domains. We believe that the strong regulation of the MS2 system combined with the flexibility and convenience of GreenGate cloning offer advantages not shared by these other systems. For example, while we mainly used the *35S* promoter for our proof-of-concept experiments, it is equally possible to use cell type-specific or environmentally inducible promoters in order to investigate the effects of targeted gene regulation under specific conditions of interest, and effector domains and sgRNAs can be combined as desired to investigate the effects of transcriptional activation, repression or various types of epigenetic modification at different sites of interest. The vector toolbox can also be expanded indefinitely to include other effector domains that were not tested here, simply by cloning the domain into the appropriate entry vector.

Furthermore, a key advantage of all CRISPR-based systems is that their RNA-guided targeting mechanism makes them highly amenable to multiplexing, which may be useful for increasing the strength of regulation by using multiple sgRNAs targeting different regions of a single gene, or for targeting multiple genes such as closely related members of a gene family or enzymes involved in consecutive steps of a metabolic pathway. This can be facilitated by using one of the various strategies that have been developed for producing multiple sgRNAs from a single transcript [39, 40], so that they can be combined into a single cloning module, or else by extending the cloning procedure so that many different sgRNAs can be incorporated independently as separate modules.

While the range of available sgRNA targets is slightly limited by the fact that the *Streptococcus pyogenes*-derived dCas9 used here requires an NGG protospacer adjacent motif (PAM) to be present in the DNA immediately adjacent to the sgRNA target site, we do not anticipate that this will be a significant constraint on target selection, as *cis*-regulatory regions tend to be broad enough to contain multiple PAM sites. However, there do exist evolved *Sp*Cas9 variants with altered PAM compatibility [41, 42], which could potentially be used with our system in place of the standard dCas9 to expand the target range and further increase the flexibility and utility of the system.

## Conclusions

The MS2-CRISPR/dCas9 vector toolkit that we have developed is effective at altering the transcriptional and epigenetic status of the Arabidopsis flowering time gene *FT*, and can be easily tailored for use with other target genes and effectors using a rapid and straightforward Golden Gate-based cloning procedure. This provides a convenient tool for investigating and manipulating the regulation of plant genes for a variety of potential purposes.

## Materials and methods

### Cloning

All plasmids were propagated using *Escherichia coli* strain DH5α and verified by sequencing. GreenGate entry and destination vectors previously described by Lampropoulos *et al*. [7] were obtained from Addgene.

#### Entry vectors

Modules for entry vectors were either synthesised *de novo*, or PCR-amplified using primers with flanking sequences containing *Bsa*I recognition sites and the appropriate overhang sequences. Phusion High-Fidelity DNA Polymerase (Thermo Scientific) was used for PCRs, and the purified PCR products were inserted into *ccdB*-containing empty entry vectors through a GreenGate reaction [7]. 9 μL of purified PCR product was mixed with 1.5 μL of the appropriate empty entry vector, 1 μL of FastDigest *Eco31*I (isoschizomer of *Bsa*I), 1.5 μL of FastDigest buffer, 1 μL of 10 mM ATP and 1 μL of 30 U/μL T4 DNA ligase for a total volume of 15 μL. Enzymes and chemical reagents were obtained from Thermo Scientific. We performed 50 cycles of digestion (37°C for 5 minutes) and ligation (16°C for 5 minutes), followed by 50°C for 5 minutes and 80°C for 5 minutes, and the entire mixture was transformed into *E*. *coli*.

For *pSUC2*, a 2.1 kb sequence upstream of the *SUC2* coding sequence was amplified from Arabidopsis genomic DNA [10]. The *SRDX* coding sequence was synthesised by overlap PCR. The *KYP* coding sequence and the C-module *RBCS* terminator were both amplified from pre-existing plasmids. The *dCas9* coding sequence was made by site-directed mutagenesis of an Arabidopsis codon-optimised version of the *SpCas9* coding sequence [43], introducing the mutations D10A and H840A. The codon-optimised *MS2* and *VP64* coding sequences and the aptamer-modified sgRNA sequences (based on sgRNA 2.0 from [4]) were synthesised by Life Technologies, and the codon-optimised *p300* and *G9a* coding sequences were synthesised by Eurofins, all with the appropriate flanking GreenGate sequences.

The sequences of primers used for cloning are listed in S2 Table, and the full sequences of all entry and intermediate plasmids can be found at Addgene under the accession numbers 122835–41, 122854–57, and 122859–65.

#### Methylated oligonucleotide duplex

Oligonucleotides with the sequences 5’-taggaccttgagacCgaaaaggtggtctCa-3’ and 5’-atactgagacCaccttttcggtctCaaggt-3’ were synthesised by Eurofins, with methyl groups on the capitalised cytosine residues, and they were annealed together by heating at 95°C for 5 minutes followed by gradual cooling.

#### Intermediate vectors

Intermediate vectors containing the *MS2-effector* cassette were assembled by GreenGate reaction [7]. 1.5 μL of each of six entry vectors (promoter, *MS2* coding sequence, effector coding sequence, dummy sequence, terminator and F-H adaptor) was mixed with 1 μL of 0.5 μM methylated oligonucleotide duplex, 1.5 μL of the *ccdB*-containing destination vector pGGZ001, 1 μL of FastDigest *Eco31*I, 1.5 μL of FastDigest buffer, 1 μL of 10 mM ATP and 1 μL of 30 U/μL T4 DNA ligase, and repeated cycles of digestion and ligation were performed as described above.

#### Final expression vectors

The final plant expression vectors were assembled in a similar fashion, by mixing six entry vectors (promoter, *dCas9* coding sequence, terminator, sgRNAs and resistance cassette) with an intermediate vector and reagents for digestion and ligation. The GreenGate reaction was performed as described above, after which any remaining intermediate vector was digested by adding an additional 0.5 μL of FastDigest *Eco31*I and incubating at 37°C for 1 hour, followed by heat inactivation at 80°C for 5 minutes. Purified plasmids were subsequently transformed into *Agrobacterium tumefaciens* strain GV3101 carrying the pSoup helper plasmid [44], for transformation into plants.

### Plant materials and growth conditions

The Columbia (Col-0) ecotype of Arabidopsis was used throughout. *ft-10* (GABI_290E08) is a T-DNA insertion mutant and has been described previously [45]. Plants were grown on soil in either LD (16 hours light/8 hours dark) or SD (8 hours light/16 hours dark) conditions, as indicated, at 22°C during light periods and 18°C during dark periods. Plant transformations were performed by floral dip [46], and transgenic plants were selected by spraying with 0.1% (v/v) Basta. Single insertion transgenic lines were chosen for further analysis in T2 and T3 generations.

### Flowering time measurement

Flowering time was measured by counting the total number of rosette and cauline leaves. In the case of SD-grown *MS2-VP64* plants with multiple shoot meristems, leaf number was counted at the time of bolting and included the basal rosette(s) only. Violin plots were generated using the BoxPlotR web tool [47].

### Fluorescence microscopy

MS2-GFP fluorescence was examined in the roots of 2-week-old plants grown on 0.5 x Murashige and Skoog medium with 0.8% agar, using a Zeiss LSM 780 confocal microscope.

### qPCR

Whole-seedling samples were collected at Zeitgeber time (ZT) 15, and RNA was extracted using the RNeasy Plant Mini Kit (Qiagen) and treated with DNase I (Thermo Scientific) to remove any contaminating genomic DNA. cDNA was synthesised from 1 μg RNA using the RevertAid First Strand cDNA Synthesis Kit (Thermo Scientific) with oligodT primers, and this was used for qPCR reactions with LightCycler 480 SYBR Green I Master (Roche Life Science) in a Bio-Rad CFX96 machine. For each sample, we used three independent biological replicates, with three technical replicates each. The sequences of primers used for qPCR are listed in S2 Table.

### ChIP

Approximately 1.5 g of whole seedlings were collected for each of three biological replicates per sample, and fixed in 1% formaldehyde for 1 hour under vacuum. ChIP was performed as described previously [48], using Abcam antibodies anti-H3K27Ac (ab4729) and anti-H3K9me2 (ab1220), at 1/500 and 1/600 dilutions, respectively. Quantification was performed by qPCR as described above and histone mark levels were calculated as enrichment in immunoprecipitated samples compared to input, normalised against either *RBCS1A* or the Cinful-like retrotransposon T5L23.29, loci that have previously been shown to carry high levels of H3K27Ac and H3K9me2, respectively [49, 50]. The sequences of primers used for qPCR are listed in S2 Table.

## Supporting information

S1 File Flowering time data for transgenic plants. (XLSX)

S1 Table Plasmids used in this study. (DOCX)

S2 Table Sequences of the primers used in this study. (DOCX)
