# Supplementary material for: CRISPR-based tools for targeted transcriptional and epigenetic regulation in plants
Source: PLoS One. 2019 Sep 26;14(9):e0222778. doi: 10.1371/journal.pone.0222778 (PMC6762090; doi:10.1371/journal.pone.0222778)
Supplement: S1 Table — (DOCX) [file pone.0222778.s002.docx]

**S1 Table. Plasmids used in this study.**

|  | **Name** | **Description** | **Overhangs** | **Bacterial resistance** | **Addgene ID** |
| --- | --- | --- | --- | --- | --- |
| **Entry vectors** | pGGA004* | *35S* promoter | A-B | Amp | 48815 |
|  | pSC142 | *SUC2* promoter | A-B | Amp | 122835 |
|  | pGGB003* | *B-dummy* (default random sequence) | B-C | Amp | 48821 |
|  | pMH220 | *dCas9* CDS | B-C | Amp | 122836 |
|  | pMH186 | *MS2* CDS | C-D | Kan | 122837 |
|  | pEF003 | *RBCS* terminator | C-D | Amp | 122838 |
|  | pGGD001* | *Linker-GFP* CDS | D-E | Amp | 48833 |
|  | pMH185 | *VP64* CDS | D-E | Amp | 122839 |
|  | pDG058 | *SRDX* CDS | D-E | Amp | 122840 |
|  | pJL023 | *p300* core domain CDS | D-E | Amp | 122841 |
|  | pJL035 | *G9a* SET domain CDS | D-E | Amp | 122854 |
|  | pJL032 | *KYP* SET domain CDS | D-E | Amp | 122855 |
|  | pMH183 | *sgRNA-FT-A* | D-E | Amp | 122856 |
|  | pGGE001* | *RBCS* terminator | E-F | Amp | 48839 |
|  | pMH184 | *sgRNA-FT-B* | E-F | Amp | 122857 |
|  | pGGF001* | *pMAS:BastaR:tMAS* | F-G | Amp | 48842 |
|  | pGGF002* | *35S:BastaR:t35S* | F-G | Amp | 48843 |
|  | pGGG001* | *F-H adaptor* | F-H | Amp | 48850 |
| **Empty destination vector** | pGGZ001* | Destination vector with counter-selectable *ccdB* gene | A-G | Spec | 48868 |
| **Intermediate vectors** | pJL003 | *35S:MS2-GFP* | A-G | Spec | 122859 |
|  | pMH204 | *35S:MS2-VP64* | A-G | Spec | 122860 |
|  | pMH205 | *pSUC2:MS2-VP64* | A-G | Spec | 122861 |
|  | pJL009 | *35S:MS2-SRDX* | A-G | Spec | 122862 |
|  | pJL024 | *35S:MS2-p300* | A-G | Spec | 122863 |
|  | pJL036 | *35S:MS2-G9a* | A-G | Spec | 122864 |
|  | pJL042 | *35S:MS2-KYP* | A-G | Spec | 122865 |
| **Final expression vectors** | pJL005 | *35S:MS2-GFP:tRBCS*  *35S:dCas9:tRBCS*  *sgRNA-FT-A*  *sgRNA-FT-B*  *35S:BastaR:t35S* | none | Spec | - |
|  | pMH211 | *35S:MS2-VP64:tRBCS*  *35S:dCas9:tRBCS*  *sgRNA-FT-A*  *sgRNA-FT-B*  *pMAS:BastaR:tMAS* | none | Spec | - |
|  | pMH212 | *35S:MS2-VP64:tRBCS*  *pSUC2:dCas9:tRBCS*  *sgRNA-FT-A*  *sgRNA-FT-B*  *pMAS:BastaR:tMAS* | none | Spec | - |
|  | pMH213 | *pSUC2:MS2-VP64:tRBCS*  *35S:dCas9:tRBCS*  *sgRNA-FT-A*  *sgRNA-FT-B*  *pMAS:BastaR:tMAS* | none | Spec | - |
|  | pMH214 | *pSUC2:MS2-VP64:tRBCS*  *pSUC2:dCas9:tRBCS*  *sgRNA-FT-A*  *sgRNA-FT-B*  *pMAS:BastaR:tMAS* | none | Spec | - |
|  | pJL011 | *35S:MS2-SRDX:tRBCS*  *35S:dCas9:tRBCS*  *sgRNA-FT-A*  *sgRNA-FT-B*  *pMAS:BastaR:tMAS* | none | Spec | - |
|  | pJL044 | *35S:MS2-p300:tRBCS*  *35S:dCas9:tRBCS*  *sgRNA-FT-A*  *sgRNA-FT-B*  *pMAS:BastaR:tMAS* | none | Spec | - |
|  | pJL049 | *35S:MS2-G9a:tRBCS*  *35S:dCas9:tRBCS*  *sgRNA-FT-A*  *sgRNA-FT-B*  *pMAS:BastaR:tMAS* | none | Spec | - |
|  | pJL050 | *35S:MS2-KYP:tRBCS*  *35S:dCas9:tRBCS*  *sgRNA-FT-A*  *sgRNA-FT-B*  *pMAS:BastaR:tMAS* | none | Spec | - |

The letters A to H indicate different complementary overhang sequences generated by *Bsa*I digestion. CDS = coding sequence.

* Lampropoulos A, Sutikovic Z, Wenzl C, Maegele I, Lohmann JU, Forner J. GreenGate - a novel, versatile, and efficient cloning system for plant transgenesis. PLoS ONE. 2013;8(12):e83043.
