# Supplementary material for: CRISPR-based tools for targeted transcriptional and epigenetic regulation in plants
Source: PLoS One. 2019 Sep 26;14(9):e0222778. doi: 10.1371/journal.pone.0222778 (PMC6762090; doi:10.1371/journal.pone.0222778)
Supplement: S2 Table — (DOCX) [file pone.0222778.s003.docx]

**S2 Table. Sequences of the primers used in this study.**

|  | **Sequence (5’ – 3’)** |
| --- | --- |
| **Primers used for entry vector cloning** | |
| pSUC2-F | AACAGGTCTCAACCTCTAAAATCTGGTTTCATATTAATTTCG |
| pSUC2-R | AACAGGTCTCTTGTTATTTGACAAACCAAGAAAGTAAGAAAAAAAAG |
| SRDX-F | AACAGGTCTCATCAGGTATGCTTGATCTCGACTTGGAGCTTAGACTCGGATTCGCTTAATAGCTGCAGAGACCTGTT |
| SRDX-R | AACAGGTCTCTGCAGCTATTAAGCGAATCCGAGTCTAAGCTCCAAGTCGAGATCAAGCATACCTGATGAGACCTGTT |
| KYP-F | AACAGGTCTCATCAGGTATGGACATCTCCGGAG |
| KYP-R | AACAGGTCTCTGCAGTTAATGAACGCTATCAAGC |
| **Primers used for Cas9 mutagenesis** | |
| H840A-F | ATGTTGATGCTATCGTGCCACAGTCATTCTTG |
| H840A-R | TGGCACGATAGCATCAACATCGTAATCAGAGAGC |
| D10A-F | ATCGGACTCGCTATCGGAACTAACTCTG |
| D10A-R | AGTTCCGATAGCGAGTCCGATAGAGTAC |
| **Primers used for qPCR and ChIP-qPCR analysis** | |
| FT-F | CCCTGCTACAACTGGAACAAC |
| FT-R | CACCCTGGTGCATACACTG |
| TUB2-F | GAGCCTTACAACGCTACTCTGTCTGTC |
| TUB2-R | ACACCAGACATAGTAGCAGAAATCAAG |
| FT1-F | TGCATGCGAAAATCTAGTGG |
| FT1-R | ACCATATGTCGCATAATGTTC |
| FT2-F | TTCGGACATTGGTAGGTATG |
| FT2-R | ACAAGGGATCCTTCAGGTTA |
| FT3-F | AATGCAAATCCGAAACAGTA |
| FT3-R | TTGAACAAACAGGTGGTTTC |
| FT4-F | TATGGTGGATCCAGATGTTC |
| FT4-R | ATTCGAGCAACATGTTTGAG |
| FT5-F | CGTAAACGACATCCATGATT |
| FT5-R | ATGCTACCTCTCCTTTTTGC |
| RBCS1A-F | CAAGCCGATAAGGGTCTCA |
| RBCS1A-R | TGATCGGAGGGTCTAGGATA |
| T5L23.29-F | GCAACCTATCAACGCTTCGT |
| T5L23.29-R | AACTCGCCAGATCTTACTCC |
